# Characteristics of Hardcore Male Smokers in Taiwan: A Qualitative Study

**DOI:** 10.31372/20200502.1085

**Published:** 2020

**Authors:** Hui-Wen Huang, Kuang-Chieh Hsueh, Wen-Wen Li, Chih-Ling Huang

**Affiliations:** a Department of Nursing, Chung Hwa University of Medical Technology, Taiwan; b b Department of Family Medicine, Kaohsiung Veterans General Hospital, Taiwan; c c School of Nursing, San Francisco State University, United States; d School of Nursing, Fooyin University, Taiwan

**Keywords:** hardcore smoker, smoking behavior, motivation to quit, public health nursing

## Abstract

*Objectives:* The purpose of this study was to explore the characteristics of hardcore smokers who were previously hospitalized. *Study design:* A descriptive qualitative design was used to investigate a sample of 29 male Taiwanese smokers. *Methods:* Male hardcore smokers were recruited at a hospital in southern Taiwan and their smoking behaviors and attitudes were explored in semistructured interviews. Inclusion criteria were: (1) smoking at least weekly or daily during the past year, (2) 100 cigarettes during the lifetime, (3) persistent smoking, or (4) at least one hospitalization. Audio-recorded interview data were analyzed using content categorization of the responses. *Results:* Four main themes emerged to describe characteristics of Taiwanese hardcore smokers: (1) physiological and psychological dependence, (2) no motivation to quit despite knowledge of negative health consequences, (3) social interaction and cultural norms, and (4) negative attitudes toward, but compliance with, smoking-free policies. *Conclusions:* The results can inform public health nurses of characteristics of Taiwanese hardcore smokers, which in turn may develop effective smoking cessation program to increase smoking cessation rate among Taiwanese hardcore smokers.

In Taiwan, the smoking rate decreased from 20.9% in 2005 to 14.0% in 2017 because of population-level tobacco control interventions (Centers for Disease Control and Prevention, 2019). This decline has recently slowed because of the supposedly irreducible number of highly nicotine-dependent smokers (i.e., hardcore smokers) who do not intend to quit (Hughes, 2011; Perez-Rios, Fernandez, Schiaffino, Nebot, & Lopez, 2015). Hardcore smokers are relatively closed to cessation interventions and have been identified as an intractable public health problem. Foreign studies have found a 13.1% to 32.2% prevalence of hardcore smokers (Bommelé et al., 2016; Kien et al., 2017). Therefore, hardcore smoking is a significant concern for public health professionals in many countries.

A “hardcore smoker” can be defined as a smoker who either is unable or unwilling to quit (Kien et al., 2017). It was reported that, in England, an average 27.9% of smokers did not want to quit, 47.6% usually smoked their first cigarette of the day within 30 minutes of waking, and 12.8% of smokers reported both (Docherty, McNeill, Gartner, & Szatkowski, 2014). One survey of hardcore smoking defined it using similar criteria (Kaleta, Usidame, Dziankowska-Zaborszczyk, Makowiec-D?browska, & Leinsalu, 2014); however, a study in the United States used the additional criteria of 26 years or older and smoking 15 or more cigarettes per day (Lam, Cheung, Leung, Abdullah, & Chan, 2015). One study defined hardcore smoking as “established daily smokers” who consume 15 or more cigarettes per day with no history of quit attempts or plans (Kang, Lee, & Cho, 2017). Another study defined it by four criteria: (1) daily smoking, (2) 15 or more cigarettes per day on average, (3) no intention to quit during the past 6 months, and (4) no attempt to quit during the previous year (Kien et al., 2017). However, defining “hardcore smoking” using these measures limits research findings because they are a limited combination of smoking behaviors (e.g., quit attempts, quit intentions, nicotine dependence, and smoking duration) (Costa et al., 2010; Lam et al., 2015).

Defining hardcore smoking is challenging and requires flexibility to fit a variety of cultural contexts. Six characteristics of hardcore smokers usually are measured: quit intention, quit attempt, nicotine dependence, long-term use, knowledge of smoking risks, and confronting substantial public disapproval of smoking (Costa et al., 2010). Dependence was the strongest predictor of persistent smoking, which seemed to offer the most potential for exploring the hardening hypothesis (Ip et al., 2012). The “hardening” idea has been applied to outcomes, such as relatively few quit attempts or a low ability to abstain/successfully stop, and to causes, such as high nicotine dependence, low motivation, low socioeconomic status, and psychiatric comorbidity. Thus, operationalizing “hardcore smoking” as a construct has been so problematic that reliable and consistent measurement across studies has not been achieved (West & Jarvis, 2018).

Research has revealed that hardcore smokers tended to have distinctive attitudes and beliefs about smoking, and social norms influenced individual-level smoking behaviors through population-level smoking-related factors (Zhang, Cowling, & Tang, 2010). Hardening might vary by population subgroup, and researchers should specifically consider group and ethnic differences (Bommelé et al., 2015). For example, smokers with certain health problems reported relatively persistent smoking (Gregor & Borrelli, 2012) and they underestimated the negative health consequences of smoking (Kaleta et al., 2014). In the Chinese context, one important challenge to addressing the hardening hypothesis has been lack of a consistent definition of a “hardcore smoker.” The lack of consensus makes it difficult to characterize persistent smokers and develop treatment recommendations for them. Thus, this study used qualitative methods to explore the personal characteristics of Taiwanese hardcore smokers who had been hospitalized.

## Methods

### Design and Sample

A descriptive qualitative research design was used to explore smoking behaviors, motivation to quit, dependence/addiction, and attitudes toward cessation. The participants were 29 adult male smokers living in southern Taiwan. Female smokers were not included because of the low proportion (2.3%) of female smokers in Taiwan (Health Promotion Administration, 2019). The participants were recruited using convenience sampling and the study was conducted from January 2016 through June 2016. Flyers were posted at a medical center inviting male smokers to participate in the study. The inclusion criteria were: (1) males aged 20 or more years, (2) smoking at least weekly or daily during the past year, (3) 100 or more cigarettes consumed during their lives, (4) at least one hospitalization, and (5) still smoking 6 months later. The exclusion criteria were: (1) a hearing impairment problem serious enough to interfere with participation and (2) any terminal illness.

### Data Collection

At the start of interview, participants completed the Chinese version questionnaire of Fagerström Test for Nicotine Dependence (FTND) which was used to confirm nicotine dependence level in hardcore smokers. This standard instrument uses six items to assess the intensity of physical addiction to nicotine. A final score is obtained by summing up the responses on yes/no items and multiple-choice items (range: 0–10), and the higher the score, the more intense the dependence (Huang, Lin, & Wang, 2006). This study yielded an internal consistency reliability coefficient (Cronbach’s of 0.71).

One 45-minute interview was conducted with each participant in the hospital and each participant received a follow-up telephone call 6 months later to determine whether he still was smoking. The 45-minute semistructured interview included questions, such as “Why do you continue to smoke?” “What do you think the association is between social interaction and smoking?” and “What do you think about the Tobacco Hazard Prevention Act?” Agee (2009) indicated that good questions in a qualitative study grow out of initial curiosity or ideas. Thus, we were based on our curiosity and ideas to develop questions of semistructured interviews rather than theory or concept framework.

All interviews were audio-recorded. The sampling of participants was concluded when qualitative data saturation was obtained. During the interviews, personal information on demographic characteristics and smoking history were collected. A semistructured interview guide was used to obtain information on the participants’ physiological and psychological smoking experiences, behaviors, positive and negative attitudes about smoking, values about smoking in public, and attitudes toward no smoking policies. The study was approved by the Ethics Review Board of the hospital (VGHKS14-CT6-01). Informed consent was obtained from the participants prior to the first interview. All participants were informed both verbally and in writing that they could withdraw from the study at any time. Confidentiality and anonymity were assured. The participants received a small gift in appreciation for their participation.

### Analysis of Questionnaire

The SPSS Version 21.0 for Windows was used for data analysis. Descriptive analyses were used to describe participants’ nicotine dependence.

### Analysis of Transcripts

The content analysis involved categorizing the qualitative responses to each question. The audio recordings were transcribed within 48 hours of the interviews. First, the interview data were synthesized by one of the researchers, who categorized the qualitative responses based on similarities and differences using ATLAS.ti (version 5) software. A summary was generated to supply details of the number of similar responses and to identify participants’ statements typical of each response category. We also made notes about topics discussed and themes that emerged to identify themes for further analysis. In the next stage, the second researcher refined the results of the initial analysis. The third researcher performed the third stage of the analysis to validate the results of the first two stages. Moreover, the second and third researchers independently evaluated the main themes and the subthemes one more time to ensure the validity of these themes. Finally, selected segments of these themes were transcribed verbatim and considered for direct quotation.

## Results

The participants were 25 to 84 years old (*M* = 50.4 years, *SD* = 14.0), smoked 20.4 cigarettes per day on average, and started smoking from 11 to 20 years old (*M* = 16.3 years, *SD* = 2.4). About one-half of the sample (*n* = 15; 51.7%) had less than a high school education; 86.2% (*n* = 25) were married and 75.9% (*n* = 22) were employed. Their colleagues smoked at work (*n* = 28; 96.6%), and only 19.3% had complete smoking bans at home (*n* = 8). The mean nicotine dependence score on the Fagerström Test or Nicotine Dependence was 4.4 (*SD* = 2.8). Most of the participants stated it was difficult to quit smoking (*n* = 24; 82.8%). Table 1 describes the results of the thematic analysis, which revealed four themes and nine subthemes. The themes were organized as “hardcore smokers who have nicotine dependence,” “do not have motivation to quit,” “perceive social interaction and cultural norms,” and “comply with smoking-free policies.”

**Table 1:** Findings: Themes and Subthemes

Theme	Subtheme
Physiological and psychological dependence	• Cravings and withdrawal symptoms
	• Self-control and feeling dependent
	• Reinforcement and habit
No motivation to quit despite knowledge of negative health consequences	• Unwillingness to quit
	• Perceived negative health consequences
Social interaction and cultural norms	• Social interaction
	• Social and cultural norms
Negative attitudes toward, but compliance with, smoking-free policies	• Ambivalent smoke-free policy
	• Compliance with smoking bans

### Theme 1. Physiological and Psychological Dependence

#### Subtheme 1: Cravings and withdrawal symptoms

The majority of participants stated that they “had cigarette cravings or withdrawal symptoms” when they did not smoke. Example responses are as follows:

*I have the habit of smoking when I wake up. Statistics say that people often smoke within a few minutes after waking up. I need to have a cigarette… I can’t stand not smoking after waking up! If I try to not smoke, I have withdrawal symptoms and can’t concentrate*.

I feel I must smoke, and I like to smoke. I also cannot sleep well without cigarettes. After drinking, I strongly feel the urge to have nicotine. When craving tobacco, I am less focused and feel anxious.

#### Subtheme 2: Self-control and feeling dependent

The most interesting result was that the majority participants stated they “depended on nicotine” or “were unable to control themselves not to smoke,” such as the following statement.

For smokers, you can only convince them to quit smoking when it is absolutely necessary. Even when they are diagnosed with a major illness, they will only quit smoking if they are told they will die if they continue smoking! For smokers, quitting smoking is like dying. From a psychological perspective, smoking is a great consolation and a way to get rid of feelings of loneliness. That is how I feel. Smoking offers me psychological comfort. When I feel bored, I naturally want to smoke. I also need to smoke when I get up in the morning. Before going to bed, I also need to have a cigarette. I don’t think I can control the craving for tobacco.

#### Subtheme 3: Reinforcement and habit

Reinforcement means hardcore smokers felt positive feedback by smoking such as keeping refreshed or concentration. Habit means smokers have regular tendency to smoke in their life.

Typical reasons for continuing to smoke were as follows:

When I feel troubled, I always want to smoke. Smoking keeps me refreshed. Particularly when I am thinking, I’ll light a cigarette, but I might not smoke it constantly. I smoke and then put it aside, and when I think of something, I’ll write it down immediately. After I finish my writing, the cigarette is gone.

*I feel weird when I do not hold a cigarette, and then I feel like having one. Smoking is a habit I have in life. I smoke when I am bored. You know your own habit*.

### Theme 2: No Motivation to Quit Despite Knowledge of Negative Health Consequences

#### Subtheme 1: Unwillingness to quit

The participants described their willingness to quit as follows:

I’ve never had the experience of stopping smoking for longer than 24 hours. I don’t want to quit smoking… I don’t know why… to be honest.

*A lot of people would think that after smoking for so long, they’d know the harm caused by smoking, but they don’t want to quit. Nowadays, we have very good health education and we all know the harmful effects of smoking on the human body, but, basically, we don’t want to quit smoking*.

My daughter does not like my smoking habit. She complains, but I do not listen to her suggestion.

#### Subtheme 2: Perceived negative health consequences

Perceived negative health consequences means hardcore smokers realized that smoking was bad for health. The majority participants told us that “smoking is not good for health.” The responses about the health risks of smoking were as follows:

*Smoking is a health issue, and I also know it’s good for your health if you don’t smoke. We know smoking causes lung cancer and oral cancer. A heavier smoker will say their chest feels tighter. If you smoke too much, you will have asthma*.

*Smoking gives you rough skin. Smoking has a negative effect on your physical strength and you will feel tired easily. We know smoking causes lung cancer and oral cancer. The most obvious effect of smoking is on your cardiovascular function because I have high blood pressure, and I know this because I’ve used myself as the subject in an experiment. I measured my blood pressure right after I smoked and my blood pressure definitely went up*.

### Theme 3: Social Interaction and Cultural Norms

#### Subtheme 1: Social interaction

Social interaction defined smoking helps smokers communicate with others and develop interpersonal relationships. Typical responses about the association between socializing and smoking were as follows:

Smoking helps you at work or social occasions probably because we have the same habit and we can communicate better. We have something in common to talk about.

Sometimes your superior asks you to smoke and chat. You can talk about business. You can smoke and talk about business at the same time. Sometimes, you also have the same hobby as your superior.

Smoking and drinking help you to develop interpersonal relationships because tobacco and alcohol are a good medium through which to help make initial contact with others.

You see many people at different ceremonies, such as weddings and funerals, smoking or asking you whether you smoke. Smoking is like a social event. If you do not smoke, you won’t feel so close to your friends.

#### Subtheme 2: Social and cultural norms

Because of these positive responses about the benefits of smoking, it was remarkable that disapproval of smoking is common in Taiwan. The participants’ comments on public attitudes and behaviors are as follows:

Even when I smoke outside, people passing by give me a dirty look or avoid me. If I light a cigarette on the street, people try to fan the air with their hand at a distance of a hundred meters away; I do not get bothered and ignore these people. For example, when I go out to pick up some snacks, I’ll smoke at the same time. If there is an old woman near me, she’ll turn her head and give me a displeased look.

When you go abroad and return to Taiwan, you will find the environment unfamiliar, as you’ll see a lot of people smoking outside the toilet at rest stations. Cigarettes are very cheap in Taiwan, but in Japan they’re very expensive. They try to increase the price to decrease the number of smokers. Smokers in Japan always have their cigarettes near an ashtray, and you won’t see smokers under the age of 18.

### Theme 4: Negative Attitudes toward, but Compliance with, Smoking-Free Policies

#### Subtheme 1: Ambivalent smoke-free policy

The participants’ comments centered on their ambivalence toward the Tobacco Hazard Prevention Act.

The government tells us not to smoke, so it should not sell cigarettes. If you do not sell tobacco, people cannot smoke. You cannot sell tobacco and tell people not to smoke at the same time. The government should not produce and sell tobacco. This is not right. Then you pay heavy taxes. This stuff is really harmful.

You sell tobacco and impose… the health and welfare surcharges, right? It doesn’t make any sense. We pay various taxes to the Taiwan government… It’s all up to the decision of the government. The health and welfare surcharges are getting higher and higher. I am also a smoker and I don’t care about the charges.

#### Subtheme 2: Compliance with smoking bans

Compliance with smoking bans means hardcore smokers’ act obeyed rules of smoking bans. The majority participants stated that they “could not smoke in a smoke-free environment.” Two responses to this question were as follows:

Smoking is not allowed in public places. I chew gum with a strong flavor to reduce my cravings for tobacco.

The Tobacco Hazards Prevention Act prohibits smoking indoors. We are in the middle and lower strata of society. We won’t go to high-end restaurants or those places where smoking is prohibited. I also cannot smoke at my workplace. Smoking at the workplace is not allowed. Now there are some non-smoking areas and, at those places, we don’t smoke.

## Discussion

To the best of our knowledge, this is the first study set in Taiwan that investigated the characteristics of hardcore smokers using qualitative methods. The results identified three main characteristics (nicotine dependence, social interaction enhanced by smoking, and compliance with smoking bans), which were similar to the characteristics of nonhardcore smokers found by previous studies (Bommelé et al., 2015; Sorg, Xu, Doppalapudi, Shelton, & Harris, 2011). The major feature of hardcore smokers was their complete unwillingness to quit, despite their awareness that smoking is harmful to human health.

### Theme 1: Physiological and Psychological Dependence

Most of the participants told us that they found it difficult to quit. Those who were physiologically and psychologically dependent on nicotine described problems with withdrawal and breaking the habit, which supports those of previous studies (Leung, Chan, Chan, & Lam, 2016; Smith, Rose, Mazure, Giovino, & McKee, 2014). Nicotine dependence is a defining aspect of hardcore smoking (Ip et al., 2012), although the relationship between smoking prevalence and the Heaviness of Smoking Index (HSI) was not significant in a study of 18 European countries (Fernandez et al., 2015). However, the HSI counts the number of cigarettes smoked per day and the duration between waking and smoking the day’s first cigarette, which might not be comprehensive enough to measure nicotine dependence (Aryal, Bhatta, Shrestha, & Gautam, 2015).

Habit and reinforcement were two reasons the participants offered for continuing to smoke, pointing out that smoking was essential to ease their stress and control unwanted emotions. However, there are ways to alleviate stress and emotional problems that are not as risky as smoking is to physical and psychological health, and healthcare professionals should help hardcore smokers find less dangerous methods.

Even with targeted education, hardcore smokers might want to continue smoking because of nicotine dependence. In our study, hardcore smokers say “I also need to smoke when I get up in the morning. Before going to bed, I also need to have a cigarette. I don’t think I can control the craving for tobacco.” Previous studies found that hardcore smokers had lower self-efficacy than nonhardcore smokers (Sorg et al., 2011), improving self-efficacy might develop smokers’ confidence in their abilities to quit (Lindberg et al., 2015), and increasing self-efficacy to resist smoking was effective for hardcore smokers (Leung et al., 2016). Therefore, a focus on increasing hardcore smokers’ self-efficacy might help them quit smoking.

### Theme 2: Social Interaction and Cultural Norms

Some of the participants pointed out advantages to smoking, such as its enhancement of social interactions. This finding supports that of von Soest and Pedersen (von Soest & Pedersen, 2014). One way that smoking might enhance socializing is through sharing cigarettes. In Taiwan, giving cigarettes to guests is a wedding custom. Smoking also might strengthen or increase personal networking and “face,” which are deeply rooted in Chinese culture (Huang, Hsueh, Lu, & Huang, 2016). Some participants told us that smoking increased their sense of social confidence and value. Smoking policy should focus on changing these customs and values. A previous study found that smokers’ perceptions of antismoking environmental norms were crucial to their smoking cessation and enforcing no smoking policies increased their motivation to quit (Chou, Chang, Kao, Lin, & Huang, 2013). Therefore, the government should publicize no smoking policies using mass media.

### Theme 3: No Motivation to Quit Despite Knowledge of Negative Health Consequences

Most of the participants stated that they knew that smoking is harmful to human health, but they did not want to quit. This is similar to a previous finding that 53.9% to 66.1% of hardcore smokers did not want to quit smoking (Kishore et al., 2013). Hardcore smokers also might have significantly more beliefs than nonhardcore smokers about the disadvantages of quitting (Bommelé et al., 2015). Moreover, some hardcore smokers think changes in body weight or social exclusion are due to quitting (Bommelé et al., 2014). Thus, hardcore smokers might have incorrect knowledge about the advantages of quitting.

Most of the participants stated that they intend to continue smoking because of their nicotine dependence and withdrawal symptoms. Nicotine replacement therapy (NRT) is one way to help them to resist nicotine cravings and prevent withdrawal symptoms (Lam et al., 2015). However, many countries fail to invest in ways to help hardcore smokers to quit (Nilan, Raw, McKeever, Murray, & McNeill, 2017). Taiwan’s government provides free NRT twice per year for smokers who intend to quit (Health Promotion Administration, 2019), but hardcore smokers tend to be exempt because they have no such intentions. Therefore, hardcore smokers in Taiwan are not being equally provided with the resources they need to stop smoking.

Public health professionals tend to design interventions to help smokers progress toward quitting using the stage of change model (Lu, Hsiao, Huang, Lin, & Huang, 2019). One previous study proposed that reducing cigarette consumption and decreasing nicotine dependence are the first steps in quitting smoking (Hsueh, Chen, Yang, & Huang, 2010). Another study indicated that clinical interventions helped unmotivated smokers to gradually move through the motivation stages toward quitting (Bani-Yaghoub, Elhomani, & Catley, 2018). Decreasing consumption also might effectively prompt cessation among hardcore smokers (Lam et al., 2015). Recently, the motivation to quit and the ability to quit were identified as separate constructs as well as key determinants for success in tobacco control efforts (West & Jarvis, 2018). Public health nurses could design interventions that encourage hardcore smokers to smoke fewer cigarettes per day to facilitate quitting. Because gradual cessation using free NRT might be effective, the government (or insurance companies) could expand the free NRT subsidy for hardcore smokers.

### Theme 4: Negative Attitudes toward, but Compliance with, Smoking-Free Policies

According to smoking-free policies in Taiwan, smoking was completely banned at public places, and smokers need to pay the health and welfare surcharges when they bought cigarettes. This study’s participants told us that the fees and taxes on tobacco products are unreasonable. Most of them complained that the government wants smokers to quit, but it still sells cigarettes. They indicated that the smoking-free policies were ambiguous. A previous study indicated that reducing the density and number of tobacco retailers may improve public health issue (Ackerman, Etow, Bartel, & Ribisl, 2017). Thus, we propose a reduction in the number of stores that sell cigarettes to persuade smokers to quit smoking.

The fees and taxes on tobacco products have consistently increased as a deterrent, but hardcore smokers are not quitting in response. These results are inconsistent with those of the World Health Organization, which concluded that increasing the costs of tobacco products is the best way to encourage cessation (World Health Organization, 2017). This study’s participants’ negative opinions of the smoking-free policies combined with further increases in tobacco taxes might produce the opposite of the desired result. Public health nurses should lobby the government to increase the tobacco tax while increasing free NRT to encourage hardcore smokers to reduce their smoking in public or quit.

Despite their resistance to quitting, we found that participants complied with the no smoking laws. A previous study found that nonhardcore as well as hardcore smokers complied with tobacco control laws/rules and that the nonhardcore smokers were relatively likely to do so (Kaleta et al., 2014). Therefore, the government might consider implementing strict enforcement of the law regarding no smoking environments.

## Limitations

Our study had some important limitations. First, the participants were limited to southern Taiwan, and hardcore smokers in other parts of Taiwan might have different smoking attitudes and behaviors. Second, although we had a 6-month follow-up to verify the smoking status of all our participants, the interview data were cross-sectional, which limits our ability to fully understand aspects of quitting among hardcore smokers because quitting smoking is a behavioral change process. A longitudinal approach would improve our knowledge. Third, because of the study’s qualitative design, predictors for intention to quit among hardcore smokers might not have been identified. Studies on female hardcore smokers and gender comparisons also would further our knowledge and inform interventions. Fourth, hardcore smokers who were Taiwanese Asians and Taiwanese immigrant may have different characteristics due to social culture. Further studies may focus on Taiwan immigration.

## Conclusions

Our study identified physiological and psychological dependence, attitudes about smoking’s link to social interaction and cultural norms, motivation to quit, and negative attitudes toward no smoking policies as major characteristics of hardcore smokers. Public health nurses should design smoking cessation programs that account for these characteristics, and Taiwan’s government should provide free NRT more often to help hardcore smokers reduce their public smoking and permanently quit.

## Acknowledgments

The authors acknowledge the funding support by Ministry of Science and Technology.

## Declaration of Conflicting Interests

The authors declared no potential conflicts of interest concerning the research, authorship, or publication of this article.

## Funding

This work was supported by Ministry of Science and Technology [grant numbers: 103-2314-B-309-002-MY3].
